# Adapting High Impact Practices in Family Planning During the COVID-19 Pandemic: Experiences From Kenya, Nigeria, and Zimbabwe

**DOI:** 10.9745/GHSP-D-22-00064

**Published:** 2022-08-30

**Authors:** Morrisa Malkin, Alexandria K. Mickler, Theophilus O. Ajibade, Alexis Coppola, Eden Demise, Esinath Derera, Joy Otsanya Ede, Meghan Gallagher, Lucia Gumbo, Zorodzai Jakopo, Kristen Little, Absolom Mbinda, Gladwin Muchena, Nyaradzo Debra Muhonde, Khesiwe Ncube, Fifi Oluwatoyin Ogbondeminu, Shannon Pryor, Elsie Nzale Sang

**Affiliations:** a FHI 360, Durham, NC, USA.; b U.S. Agency for International Development/Public Health Institute, Washington, DC, USA.; c Society for Family Health, Abuja, Nigeria.; d Population Services International, Washington, DC, USA.; e FHI 360, Harare, Zimbabwe.; f Save the Children, Washington, DC, USA.; g U.S. Agency for International Development, Harare, Zimbabwe.; h FHI 360, Mutare, Zimbabwe.; i FHI 360, Bulawayo, Zimbabwe.; j Save the Children International, Nairobi, Kenya.

## Abstract

**Background:**

We describe how High Impact Practices (HIPs) in family planning (FP) were adapted across Kenya, Nigeria, and Zimbabwe to maintain access to services in response to the coronavirus disease (COVID-19) pandemic.

**Methods:**

Using a qualitative data collection tool structured around 3 HIP categories (service delivery, demand creation, and enabling environment), adaptations in FP programs during the pandemic were documented. We describe adaptations made to 3 specific HIPs: mobile outreach, community health workers, and digital health for social and behavior change.

**Program Experiences:**

In Zimbabwe, the Mhuri/Imuli project adapted its mobile outreach model integrating community-based outreach with facility-based outreach. The number of outreach clients served per week peaked at 1,759 (July 2020) from a low of 203 (May 2020). Clients choosing long-acting reversible methods increased from 22% to 59% during the 3 months before and after lockdown, respectively.

In Kenya, a program addressed youth’s hesitation to visit health facilities through youth community health volunteers, who provided counseling, community dialogues, contraceptive pills, and condoms. Over 6 months, the program reached 1,048 youth with community dialogues, and 4,656 youth received FP services. In Nigeria, peer mobilizers provided services through a socially distanced community-based program to help adolescent girls access contraceptive self-injection when movement restrictions limited youth’s ability to travel to facilities.

In Nigeria, Adolescents 360 adapted sexual and reproductive health information programs for virtual delivery through WhatsApp. A contraceptive education Facebook campaign gained more than 80,000 followers, reached 5.9 million adolescents, and linked 330 adolescents to program-supported facilities from January to March 2021. In Kenya, the Kibera-based project used WhatsApp to reach youth with discussion groups and health workers with skills strengthening.

**Conclusion:**

Monitoring how projects adapt HIPs to ensure continuity of care during the COVID-19 pandemic can help inform the implementation of successful adaptations in the face of present and future challenges.

## Abstract (precis)

Documenting how family planning programs adapt to ensure continuity of care during the COVID-19 pandemic is an important contribution toward implementing approaches that are effective and resilient in the face of present and future challenges.

## Teaser

**Key Findings:**

- The documented adaptations primarily consisted of small adjustments to existing activities and approaches in direct response to challenges posed by COVID-19.
- Across each High Impact Practice (HIP), the ability of projects to be flexible and quickly respond to challenges as they arose supported continuous service delivery and helped meet family planning clients’ needs.

**Key Implications:**

- Program managers should disseminate the lessons learned from implementing these adaptations, as well as seek to incorporate them into resources such as planning and preparedness guides. Where synergies exist, the lessons learned may be packaged with those of similar efforts to maximize the application of learnings.
- Program managers and national stakeholders should use these and other learnings from the global community of practice to inform continued implementation of HIPs as the global health community works to evolve approaches to become more effective and resilient in the face of present and future challenges.

## INTRODUCTION

The coronavirus disease (COVID-19) pandemic has greatly impacted global health systems, including disruptions in sexual and reproductive health (SRH) programs, disproportionally affecting women in low- and middle-income countries.^1^^–^^4^ A survey by the World Health Organization found that nearly 70% of the 105 countries surveyed experienced family planning (FP) service disruptions due to the pandemic, which may result in increased maternal, newborn, and child morbidity and mortality.^5^ In a study in Nepal, Niger, Malawi, and Uganda, more than three-quarters of women reporting unintended pregnancies indicated that the pandemic had affected their ability to delay or avoid getting pregnant. Data from multiple sub-Saharan African countries indicated an increased need for contraception among nulliparous women and women with pandemic-induced income loss.^3^^,^^6^^,^^7^

These inequities highlight the need for FP programs to continuously adapt and implement evidence-based practices to mitigate service disruptions in light of lockdowns, supply chain issues, and other secondary pandemic effects.^8^^,^^9^ During the pandemic, many projects developed initiatives to track the impact of COVID-19 on global FP programs.^11^ In response to the challenges of the COVID-19 pandemic, 3 FP projects implemented and adapted select High Impact Practices (HIPs) in FP: mobile outreach services, community health workers, and digital health for social and behavior change (Box).^10^ We discuss key takeaways that program implementers may consider applying to address similar challenges and structure programming to be resilient to future crises.

BOX **Categories of High Impact Practices in Family Planning**

The High Impact Practices (HIPs) are a set of evidence-based practices that are identified through ongoing, thorough reviews of the peer-reviewed and programmatic literature and are prioritized based on their impact on multiple reproductive health outcomes.^10^ The HIP Partnership develops HIP knowledge management products, including HIP briefs, organized across 3 categories:

- **Service delivery HIPs** improve the availability, accessibility, acceptability, and quality of family planning (FP) services. We discuss projects that adapted the following HIPs:
  - **Support mobile outreach service delivery to provide contraceptive methods:** Mobile outreach models allow for flexible and strategic deployment of necessary FP resources, including health care providers, FP commodities, supplies, equipment, and more to areas in greatest need. By addressing access barriers, mobile outreach can broaden the availability of contraceptives by bringing information, services, and supplies to where women and men live and work.
  - **Integrate trained, equipped, and supported community health workers (CHWs) into the health system**: By integrating trained, equipped, and supported CHWs into the health system, FP programs can increase the availability and use of contraception, particularly in areas where geographic, social, or other barriers prevent access to FP information and services. CHWs can provide a range of services, including health education, referral and follow-up, preventive health care, home visiting services, and other skills depending on their level of training and education.
- **Social and behavior change HIPs** influence knowledge, beliefs, behaviors, and social norms associated with FP. We discuss projects that adapted the following HIP:
  - **Use digital technologies to support healthy sexual and reproductive behaviors**: Using digital technologies to convey information and messages as part of an evidence-based social and behavior change strategy can contribute to the maintenance and adoption of healthy sexual and reproductive health behaviors. Digital technology may include mobile phones, computers, or tablets, and its use may contribute to shifting norms, influencing attitudes and behaviors, and increasing self-efficacy and reproductive health knowledge.
- **Enabling environment HIPs** address systemic barriers that affect an individual's ability to access FP information and services.

FP programs need to continuously adapt and implement evidence-based practices to mitigate service disruptions in light of lockdowns, supply chain issues, and other secondary pandemic effects.

## METHODS

After COVID-19 was declared a global pandemic, the Research for Scalable Solutions project, funded by the U.S. Agency for International Development, developed an Excel-based qualitative data collection tool (Supplement) to systematically document adaptations designed, implemented, and monitored by local project teams across 8 projects implemented by Research for Scalable Solutions partners in India, Kenya, Nigeria, Uganda, and Zimbabwe. The tool was structured around 3 HIP categories (Box) and was designed to capture how program teams operated, adapted, and made decisions in a crisis situation. Every month, project teams populated the tool by documenting planned activities, pandemic-related challenges, and any adaptations made in response to the challenges. Project teams also conducted secondary data analyses of routine FP process and outcome indicators and documented policy environment changes at the country level.^12^

To best demonstrate the selected HIPs, we highlight adaptations made by 3 projects: Mhuri/Imuli in Zimbabwe, Adolescents 360 (A360) in Nigeria, and Strengthening Access to Adolescent Sexual and Reproductive Health Services during COVID-19 in Kenya. The documentation period for Mhuri/Imuli and A360 spanned from April through November 2020. The documentation period of Strengthening Access to Contraception among Adolescents during the COVID-19 pandemic was July through December 2020 to accommodate that project’s start date.

## Program EXPERIENCES IMPLEMENTING HIPs

We describe 3 FP projects that implemented and adapted the following HIPs in FP: (1) support mobile outreach service delivery in Zimbabwe; (2) integrate trained, equipped, and supported community health workers into the health system in Kenya and Nigeria; and (3) use digital technologies to support healthy sexual and reproductive behaviors in Nigeria.

### Mobile Outreach Services Maintain FP Access in Zimbabwe

The first case of COVID-19 in Zimbabwe was reported on March 21, 2020, followed by a stringent lockdown on March 30. Simultaneously, the country was struggling with a malaria outbreak, exacerbating the effects on the health care system including shortages of personal protective equipment and recurring health worker strikes throughout the year.

The USAID-funded Mhuri/Imuli project, led by FHI 360, was in its third year of supporting the Ministry of Health and Child Care (MOHCC) and the Zimbabwe National Family Planning Council to improve maternal, newborn, and child health, as well as FP services in Zimbabwe. Mobile outreach was central to Mhuri/Imuli’s approach, which involved teams of clinicians traveling to hard-to-reach communities to offer FP services. However, in April 2020 as part of the government’s COVID-19 response, outreach was temporarily suspended. Demand for FP services also decreased, especially during the early days of the pandemic when misinformation about how the virus spreads and how to prevent it was pervasive on social media and within communities. Fear of COVID-19 transmission, lockdowns, curfews, movement restrictions, and lack of public transport led to people’s reduced use of services, even if they were desired or needed. There was also a lack of clarity on how to access services considering frequently changing restrictions. In response, the project developed a fact sheet on the importance of FP during the COVID-19 pandemic. The fact sheet included messages on the impact of COVID-19 on FP, why FP is an essential service, that FP services continue to be available, and safe ways of accessing FP services during the pandemic. The messages were shared verbally with FP clients by outreach teams during group health education sessions.

Before the COVID-19 pandemic and the temporary suspension of outreach services, the Mhuri/Imuli project only provided outreach services in communities, away from health facilities. However, facilities were experiencing challenges providing FP services as the number of staff permitted to be on duty at the same time was reduced and staff’s focus shifted to providing emergency services only. In addition, some facilities did not have any staff trained to provide long-acting reversible contraceptive methods (LARCs). The combination of these factors meant considerable disruption of FP service delivery and decreased method choice. In response, Mhuri/Imuli implemented a modified outreach model that involved stationing outreach staff at health facilities to offer FP services including implants (Implanon NXT and Jadelle), copper intrauterine devices, oral contraceptive pills, injectables, male condoms, and female condoms. In facilities where staff were available to provide short-term methods but not LARCs (due to lack of training or staff shortages), Mhuri/Imuli focused on LARC provision. To support this model, community health volunteers (CHVs) and FP service providers directed clients to facilities when possible, while raising awareness of FP. The project adapted this mixed model as a best practice even when community outreach resumed in June 2020, as this approach gave MOHCC nurses time to focus on additional duties imposed by COVID-19.

*We were scared of the unknown, but we knew there were people out there in the hard-to-reach areas who needed our services, and this gave us strength and courage. It could have been risky, but the services were essential. With required [personal protective equipment] support and knowledge, we continued to offer FP services.* —Mashonaland East Team Leader, Mhuri/Imuli Project, Zimbabwe

**Figure:**
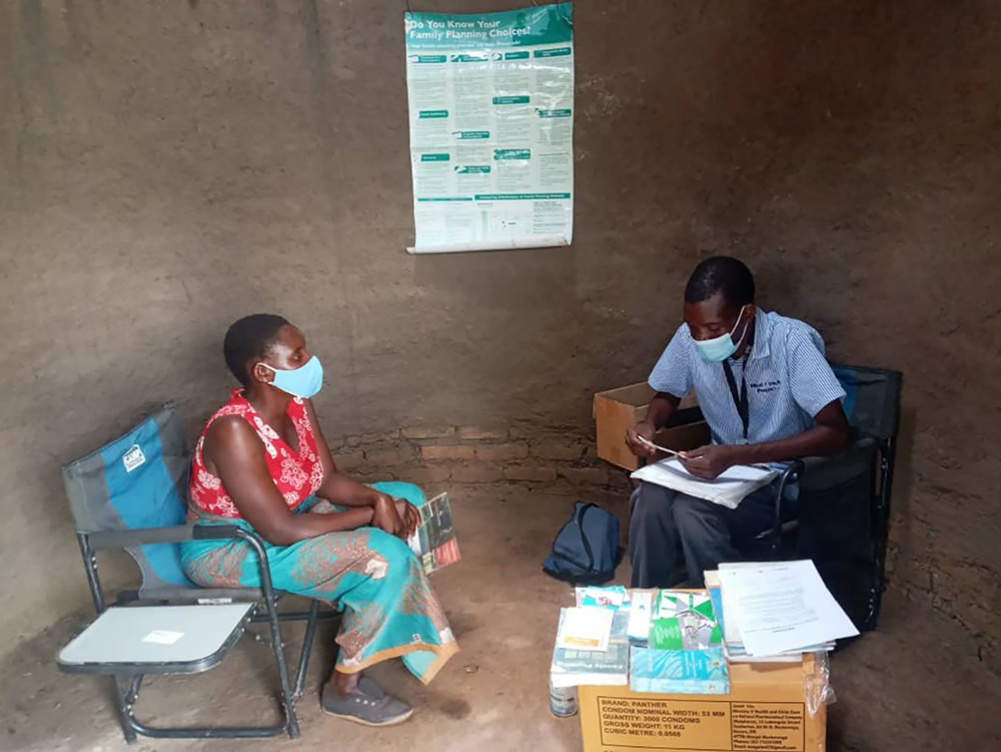
Mashonaland West Team Leader counsels a client on family planning at a community point in Gambuli village, Makonde, Zimbabwe. © 2021 FHI 360

In response to pandemic-related challenges, Mhuri/Imuli implemented a modified outreach model that involved stationing outreach staff at health facilities to offer FP services.

Before the COVID-19 pandemic, Mhuri/Imuli outreach teams provided integrated FP services as part of some community-level MOHCC immunization activities. However, when outreach was suspended and movement restricted, the project greatly expanded this model to provide FP services with other health services, including cervical cancer screening and antiretroviral therapy distribution across 9 provinces in partnership with Plan International, I-TECH, and the Ministry of Women Affairs. Integrating with other health services enabled the project to continue providing FP services when it may not have otherwise been possible and allowed women to access more services at 1 time to reduce their contact with an overburdened health system.

Documentation occurred from April to November 2020 and showed the number of outreach clients served in 1 week peaked in July 2020 at 1,759 (from a low of 203 in May 2020). During the 3-month periods before and after lockdown, the modified outreach model led to an increase in clients choosing LARCs from 22% to 59%, respectively. The uncertainty associated with intermittent lockdowns and clients’ desire to reduce the frequency of visits to clinics were some of the reasons behind the increased uptake of LARCs. Also, the modified outreach model helped ensure the continuity of FP services at facilities, thereby allowing clients the ability to exercise method choice. The project continues to station outreach teams at static health facilities to complement community-based outreach, which has resumed.

### Youth Community Health Volunteers Reach Young Adolescents in Kenya

In Kibera, Kenya, after the first case was detected in March 2020, a partial lockdown was enacted that included movement restrictions, containment (restricted entry in and out of Nairobi), and curfews. Save the Children, which had been operating in Kibera before the pandemic, sought funding from a private donor to maintain access to contraceptive services and SRH information for adolescents (aged 10–19 years).

Recognizing clients’ concerns about seeking facility-based services during the COVID-19 pandemic and cognizant that adolescents may not be comfortable accessing FP services from older, adult CHVs who traditionally provided community-based FP services, the Strengthening Access to Adolescent Sexual and Reproductive Health Services during COVID-19 project recruited a cadre of youth CHVs who were closer in age to the clients they served. Youth CHVs were recruited and connected to specific health facilities where they submitted data reports and received commodity resupply. They were trained on FP and basic contraceptive method counseling and paired with existing CHVs who served as mentors. For safety, masks were worn during interactions, and training activities were limited to 12 participants.

Recognizing that adolescents may not be comfortable accessing FP services from adult CHVs, the project recruited and trained youth CHVs who were closer in age to the clients they served.

Youth CHVs provided counseling and distributed pills and condoms. In addition, they mobilized and/or led community dialogues and small group discussions with adolescents* on topics including body literacy and fertility awareness, menstruation, preventing unintended pregnancy, healthy timing and spacing of pregnancy, nutrition, pregnancy, and parenting. After hearing anecdotal reports of increasing pregnancy rates among very young adolescents (10–14 years), youth CHVs held discussion groups for pregnant or parenting very young adolescents to discuss what to expect during pregnancy and delivery, care for the newborn, and spacing subsequent pregnancies. The youth CHVs were able to relate to young people seeking FP and create opportunities to discuss FP freely, without the stigma that often existed previously with adult CHVs.

In the 30 participating facilities, the number of adolescents and youth (aged 15–24 years) receiving contraceptive services from July to December 2019 was 4,245. From July–December 2020, 4,656 adolescents and youth received services from the same facilities, showing no decline during the documentation period.

### Peer Mobilizers in Nigeria Help Adolescents Access SRH Services

In Nigeria, A360, a project funded by the Bill and Melinda Gates Foundation and the Children’s Investment Fund Foundation and implemented by the Society for Family Health, aimed to increase the voluntary uptake of modern contraceptives among adolescent girls ages 15–19 years. In May 2020, when COVID-19 movement restrictions limited youth’s ability to travel to facilities and access SRH services, A360 fast-tracked “Big Sistas,” a pilot community-based program to distribute self-injection of subcutaneous depot medroxyprogesterone acetate (DMPA-SC) and adapted it to adhere to social-distancing restrictions. Big Sistas are experienced, knowledgeable peer mobilizers who are satisfied DMPA-SC users based in the community and could relate to the experience of choosing a contraceptive method. Big Sistas were tasked with training, referring, supplying, and supporting other adolescents interested in DMPA-SC through one-on-one counseling, following social distancing protocols. Medically trained Big Sistas could initiate adolescent girls on DMPA-SC or train them to self-inject, while not medically trained Big Sistas were to refer girls to a provider for their first injection.

Between May and June 2020, 25 Big Sistas were trained, 152 adolescents initiated DMPA-SC, and 40 adolescents were referred. Despite the challenges of a pandemic context, A360 saw a 38% increase in married and a 100% increase in unmarried adolescent girls within the A360 network who adopted DMPA-SC for the first time between May and June 2020. Although not an official cadre of community health workers, Big Sistas acted as a trusted and direct channel for clients to access DMPA-SC and self-injection outside of the facility setting, while creating linkages between facilities, providers, and clients through community awareness and community-based distribution of SRH commodities. Given this success, A360 continues to scale this program by training new peer mobilizers every month and retaining their existing network of Big Sistas in Southern Nigeria, including replicating this model for A360’s programming in Northern Nigeria.

### Digital Adaptations in Nigeria Maintain Access to SRH Information

After COVID-19 emerged in Nigeria in February 2020, the country imposed a lockdown of nonessential activities, closure of schools, ban on international flights, and curfew.^13^ In its fourth year of implementation at the time, A360 Nigeria faced significant challenges to safely provide SRH information through the in-person Life, Love, and Health (LLH) classes and SRH services at A360-supported facilities in 5 states. The LLH curriculum offers information to adolescent girls on vocational skills, love, relationships, and health to generate demand for contraceptive uptake. Safety concerns coupled with government-imposed curfews and lockdowns in Southern Nigeria caused the program to suspend in-person LLH classes.

In May 2020, in response to COVID-19 movement restrictions, A360 began digitizing the LLH classes via WhatsApp to safely reach adolescent girls with relevant SRH information and linkages to products and services. The WhatsApp LLH classes were offered in a group chat format by geographic clusters and included the option of one-on-one counseling with a provider who could refer and link girls with SRH facilities. Between May and October 2020, A360 created 40 WhatsApp groups, enrolled approximately 2,000 girls in the groups and held 26 class sessions. The girls enrolled had opportunities to ask questions during the group sessions and were linked to a provider for confidential follow-up and referrals to SRH products and services. The WhatsApp platform helped build trust and confidence. After curriculum digitization (as well as other adaptations including the inclusion of 34 additional spoke clinics in May 2020) modern contraceptive method uptake among girls within the A360 network increased by 130%, from 2,260 in May 2020 to 5,202 in June 2020, and remained at similar levels through August 2020. Given the success of the WhatsApp LLH classes in Southern Nigeria, the curriculum was also expanded to A360’s programming in Northern Nigeria.

Among girls within the A360 network, modern contraceptive method uptake increased 130% from May to June 2020 after curriculum digitization and other adaptations.

In April 2020, A360 also revamped the Facebook page for 9ja Girls, a program that provides information and linkages to SRH products and services, and revitalized their communication plan to reach more girls. As a result, the 9ja Girls page saw an organic growth of 9,600 additional followers (growth from 2,400 to 12,000) between April and September 2020.

Seeing the potential for increased reach, A360 launched a new contraceptive education Facebook campaign and developed new partnerships with Blu Flamingo for content creation and UpSwell for technical support. Between October 2020 and March 2021, the Facebook campaign reached 5.9 million adolescent girls and gained 85,866 additional followers. Between January and March 2021, the campaign linked 330 girls to contraceptive counseling and referrals. A360 continues to explore partnerships with other non-A360 supported facilities to further facilitate contraceptive uptake and with additional online retailers offering over-the-counter SRH products to support contraceptive method continuation for girls without access to a provider.

*Because of the potential that we’ve seen with digital solutions in creating demand, we are moving forward with both the WhatsApp Group and Facebook Campaign. For Facebook, we are creating linkages to online markets where they are able to access over-the-counter contraceptive methods.* —Joy Otsanya Ede, A360 Project, Society for Family Health, Nigeria

### Digital Meetings in Kenya Complement In-Person Discussion Groups

Save the Children’s project in Kenya utilized digital adaptations to continue reaching youth with discussion groups during group gathering restrictions. While in-person discussion groups were limited to 12 participants and the frequency was limited to monthly meetings, the project used WhatsApp discussion groups to complement the in-person discussion groups to maintain the reach and momentum of discussions. Trained adult CHVs facilitated the discussions that included information sharing, question and answer sessions, weekly prompts to spark discussion, and the use of short vignettes. While the WhatsApp discussion groups did not replace in-person groups and the project did not provide data for participation, phone ownership among adolescents in Nairobi is high and the virtual discussion groups kept the groups active. This allowed the project to continue discussions given less frequent in-person contacts, engaged additional young people, and allowed CHVs to build stronger ties with the youth community.

The project in Kenya used WhatsApp discussion groups to complement the in-person discussion groups to maintain the reach and momentum of discussions.

In addition to the WhatsApp discussion groups for young people, health workers also participated in virtual facilitated WhatsApp groups intended to strengthen their skills and confidence in providing SRH services to young people. The approach used vignettes of potential service delivery scenarios to generate discussion among health workers and reinforce best practices. This approach will be further tested through the Protect project in 2022.

## KEY TAKEAWAYS

As projects adapted to ensure SRH services continued during the pandemic, a few key takeaways emerged.

- **Mobile outreach is an effective approach for delivering FP services:** Mobile outreach is both a HIP and considered to be an intervention that should be prioritized when facility-based SRH services are disrupted.^14^ Our experiences in Zimbabwe reinforce mobile outreach as an effective approach for delivering FP services, especially in situations where access to trained health providers is limited. Moreover, the flexibility of mobile outreach services was especially applicable to the dynamic nature of COVID-19 restrictions in Zimbabwe. By offering FP outreach services at facilities and integrating them into other services, the project continued serving clients when it may otherwise have experienced a protracted gap in service delivery.
- **Community health workers are an effective way to reach clients when and where access is limited:** Delivery of FP services by trained, equipped, and supported community health workers is a HIP and prepandemic mainstay of many FP programs. The project experiences in Kenya with youth CHVs and Nigeria with Big Sistas further demonstrated that they can continue to be an effective (and in many cases critical) channel of service delivery in the face of COVID-19 challenges. The existing presence of community health workers within the communities they serve means that they can reach clients less hindered by challenges such as movement and gathering restrictions, as shown through the one-on-one engagement between Big Sistas and adolescents within the A360 project.
- **Digital technologies can help youth and adolescents access SRH information and services**: Lastly, digital interventions, already expanding in use before the pandemic, became a critical component of projects as protracted lockdowns and movement restrictions limited the use of more traditional, in-person strategies for reaching clients and supporting providers. The use of digital technology became a crucial social and behavior change strategy that was particularly suited to projects focused on reaching youth populations as these approaches demonstrated that they maintain demand and access to SRH services. The 9ja Girls campaign in Nigeria demonstrated social media’s potential to reach a wider range of youth both during and beyond a pandemic setting. Lastly, digital interventions that do not have additional fees beyond data usage (such as those that are social media-based), may be accessible and economical for certain populations.

Two cross-cutting takeaways also emerged.

- **Adaptations were smaller, localized adjustments**: The adaptations we documented primarily consisted of small adjustments to existing activities and approaches in direct response to a given challenge. Large, sweeping changes were generally not necessary, even in the context of this unprecedented global crisis. Adaptations were also highly localized to their own contexts. For example, Save the Children adapted its approach in light of increasing pregnancy rates among very young adolescents by creating discussion groups tailored specifically for pregnant or parenting very young adolescents.
- **Adaptations will be ongoing in the face of evolving challenges:** Mhuri/Imuli’s outreach teams joining with the Ministry of Health and other partners to provide services at the community level, Save the Children using WhatsApp to reach youth despite gathering restrictions, and A360 pivoting from in-person LLH classes to delivering content virtually are all examples of projects making deliberate, rapid changes to their approaches in the face of extreme challenges. However, it is important to continuously monitor and assess adaptations to ensure they are still meeting the needs of the moment. As pandemic restrictions evolved, adaptations also had to evolve. For example, as restrictions on in-person gatherings were reduced, the limitations of WhatsApp discussion groups prompted Save the Children to invest more in the in-person groups, making sure they could be conducted safely. At the same time, the pandemic reinforced the importance of certain services, such as mobile outreach, while providing an opportunity to realize the benefits of adapting this approach to the facility context.

As pandemic restrictions evolve, it is important to continuously monitor and assess adaptations to ensure they are still meeting the needs of the moment.

## CONCLUSIONS

The projects included in this article were diverse in terms of the contexts and populations they served, as well as in their approach to the HIPs. Despite these differences, all adaptations were implemented with the goal of ensuring continuity and quality of FP services; adhering to national and local guidelines; and maintaining the safety of clients, communities, and project staff. While tracking the costs associated with the adaptations was not part of this activity’s scope, some adaptations incurred unanticipated expenses, such as the need to hold additional trainings when the number of participants was limited. For other adaptations, projects realized cost savings which then allowed for redistribution of funds to new approaches. All 3 projects, however, were able to operate within their allocated funding. Future efforts to track the costs associated with adapting FP programs to a pandemic context would contribute to the knowledge base and help programs be better prepared for potential emergencies.

Throughout the pandemic, projects gained critical experience and learned lessons that are informing which adaptations can and should be continued even when the challenges of the COVID-19 pandemic are felt less acutely. Some adaptations may no longer be needed, while others revealed opportunities for improved service delivery, more equitable access, or enhanced reach. Documenting and learning how HIPs are adapted to ensure the continuity of care during the COVID-19 pandemic is a critical contribution to our global learning and provides insights into the effectiveness of adaptations and the value of permanently incorporating adaptations into programming.

While it will be important to use these and other learnings from the global community to inform continued implementation and ensure the quality and resiliency of FP programs during this pandemic, COVID-19 is not the only challenge facing reproductive health and global health more broadly. Humanitarian crises, natural disasters, and systemic structural inequities constitute a global shift that illuminates what aspects of global health efforts have failed and what aspects are successful. Both experiences are equally important to share as the global health community collectively evolves to become more effective, adaptable, and resilient for future challenges.

## Supplementary Material

GHSP-D-22-00064-supplement.xlsx

## Footnotes

* Informed by several systematic reviews, peer education programs have been found to result in information sharing but have limited effects on promoting healthy behaviors. Youth CHVs go beyond peer education as they perform the CHW function providing information, counseling, and services.

## Notes

**Peer Reviewed**

**First published online**: August 18, 2022.
